# Reproducibility of retinal and choroidal measurements using swept-source optical coherence tomography in patients with Parkinson’s disease

**DOI:** 10.5935/0004-2749.20200008

**Published:** 2020

**Authors:** Javier Obis, Elena Garcia-Martin, Elvira Orduna, Elisa Vilades, Raquel Alarcia, Maria J Rodrigo, Luis E. Pablo, Vicente Polo, Jose M. Larrosa, Maria Satue

**Affiliations:** 1 Ophthalmology Department, Miguel Servet University Hospital, Zaragoza, Spain; 2 Miguel Servet Ophthalmology Innovative and Research Group, Aragon Institute for Health Research, Zaragoza, Spain; 3 Neurology Department, Miguel Servet University Hospital, Zaragoza, Spain

**Keywords:** Retina, choroid, Parkinson’s disease, Optical coherence tomography, Retina, Coroide, Doença de Parkinson, Tomografía de coerência óptica

## Abstract

**Purpose:**

To assess the reproducibility of retinal and choroidal measurements in the macular and peripapillary areas using swept-source optical coherence tomography in patients with Parkinson’s disease.

**Methods:**

A total of 63 eyes of 63 patients with idiopathic Parkinson’s disease were evaluated using a three-dimensional protocol of swept-source optical coherence tomography. The following layers were analyzed: full retinal thickness, retinal nerve fiber layer, ganglion cell layer, and choroid. The coefficient of variation was calculated for every measurement.

**Results:**

In the macular area, the mean coefficients of variation of retinal thickness, ganglion cell layer + thickness, and choroidal thickness were 0.40%, 0.84%, and 2.09%, respectively. Regarding the peripapillary area, the mean coefficient of variation of the retinal nerve fiber layer thickness was 2.78. The inferior quadrant showed the highest reproducibility (coefficient of variation= 1.62%), whereas the superonasal sector showed the lowest reproducibility (coefficient of variation= 8.76%).

**Conclusions:**

Swept-source optical coherence tomography provides highly reproducible measurements of retinal and choroidal thickness in both the macular and peripapillary areas. The reproducibility is higher in measurements of retinal thickness versus choroidal thickness.

## Abstract (pt)

**Objetivo:**

Avaliar a reprodutibilidade das medições da retina e da coroide nas áreas macular e peripapilar utilizando a tomografia de coerência ótica com fonte de varredura pacientes com doença de Parkinson.

**Métodos:**

Um total de 63 olhos de 63 pacientes com doença de Parkinson idiopática foram avaliados usando um protocolo 3D de tomografia de coerência ótica de fonte Triton Swept. Foram analisadas as seguintes camadas: espessura retiniana total, camada de fibras nervosas da retina, camada de células ganglionares e coróide. O coeficiente de variação foi calculado para cada medição.

**Resultados:**

Na área macular, os coeficientes médios de variação da espessura da retina, da camada de células ganglionares + espessura e da espessura da coróide foram de 0,40%, 0,84% e 2,09%, respectivamente. Em relação à área peripapilar, o coeficiente médio de variação da espessura da camada de fibras nervosas da retina foi de 2,78%. O quadrante inferior apresentou a maior reprodutibilidade (coeficiente de variação= 1,62%), enquanto o setor superonasal apresentou a menor reprodutibilidade (coeficiente de variação= 8,76%).

**Conclusões:**

A tomografia de coerência ótica de fonte Triton Swept fornece medições altamente reprodutíveis da espessura da retina e da coroide nas áreas macular e peripapilar. A reprodutibilidade é maior nas medidas da espessura da retina versus a espessura da coróide.

## INTRODUCTION

Parkinson’s disease (PD) is a neurodegenerative process causing selective loss of dopaminergic neurons^(1)^. The prevalence of PD is approximately 0.3% in those aged >40 years, implying that 7.5 million individuals suffer from the disease worldwide^(2)^.

PD causes motor alterations, such as bradykinesia, resting tremor, rigidity, or postural instability^(3)^. Non-motor symptoms include autonomic dysfunction, depression, or dementia^(4)^. Additionally, PD affects vision, especially the visual field corresponding to the fovea^(5)^. Since 2004, a large number of studies have demonstrated alterations in macular thickness, including variable results depending on the different layers and areas. Moreover, decreased thickness of the retinal nerve fiber layer (RNFL) in the peripapillary area has been noted, especially in the temporal and inferior quadrants^(6^-^9)^. Thus far, there are only a few studies investigating the choroidal thickness in PD patients, with contradictory results^(10^,^11)^.

In recent years, optical coherence tomography (OCT) has achieved considerable progress. The emergence of swept-source OCT (SS-OCT) has improved the evaluation of the retina and choroid versus the previously used Fourier-domain OCT (FD-OCT). SS-OCT devices utilize longer wavelengths compared with those used for FD-OCT (i.e., 1,050 nm versus 840 nm, respectively). Thus, SS-OCT involves less light scattering on the choroid and obtains a faster scan speed of up to 100,000 A-scans/second. This produces more precise images of the retina and choroid^(12)^. Moreover, in SS-OCT, the limits of the layers are automatically defined. In contrast, in FD-OCT, these limits are manually established by the operator in each case.

In statistics, the coefficient of variation (COV) is used to assess the reproducibility of a parameter. The COV of a parameter is expressed as a percentage, and is calculated as the standard deviation divided by the mean value of that parameter and multiplied by 100. The COV assesses the variability of a parameter more accurately compared with the standard deviation. Notably, the reproducibility of a parameter is negatively associated with the COV. Measurements with a COV <10% are considered highly reproducible, whereas those with a COV <5% are considered highly reproducible^(7)^.

Thus far, only one study, utilizing two FD-OCT devices to evaluate the peripapillary area, investigated the reproducibility of OCT technology in PD^(7)^.

Recent studies, using SS-OCT devices, have evaluated the reproducibility of retinal measurements in macular pathologies^(13)^ and the insertion distance of rectus muscles^(14)^. Another recent study used automated SS-OCT to evaluate the thickness of the choroid and RNFL in eyes with nonarteritic anterior ischemic optic neuropathy. The study compared these eyes with contralateral unaffected eyes and healthy control eyes^(15)^. However, currently, there are no studies investigating the reproduci bility of SS-OCT in PD, or that of choroidal measurements using SS-OCT technology in pathological or healthy eyes. In this study, we assessed the reproducibility of macular and peripapillary measurements in PD obtained using a SS-OCT device to evaluate different retinal layers and the choroid through automated layer segmentation.

Previous studies have examined the eyes of patients with PD using FD-OCT and SS-OCT. The investigators concluded that retinal and choroidal thickness, measured using both methods, may be a non-invasive biomarker for PD^(7^-^9)^.

The different layers of the retina are specifically affected by neurodegeneration in PD^(7^-^9)^. Therefore, it is important to independently and accurately measure the thickness of these layers. This can be performed through automated segmentation using SS-OCT.

Changes in retinal and choroidal measurements in the same individuals over a period time may be attributed to the presence of progressive thinning or thickening. Additionally, changes may be caused by the variability of the device utilized to perform the measurements. For example, evaluation of the inferior area of the RNFL in one eye of a patient with PD using an OCT device has yielded a measurement of 125 microns, with variability of 1.5 microns. One year later, a measurement performed in the same area using the same device yielded a measurement of 120 microns. Based on these finding, it can be concluded that the difference of 5 microns may be due to thinning caused by neurodegeneration. However, if the variability of the device in that particular area is 6 microns, the observed difference (i.e., 5 microns) may be due to that variability rather than thinning. Consequently, assessing the reproducibility of the device is of crucial importance to determine the degree of variability.

The aim of this study was to assess the applicability of SS-OCT in clinical practice for the evaluation of patients with PD.

## METHODS

This study included patients with idiopathic PD. The diagnosis of PD was reached by one experienced neurologist using the United Kingdom Brain Bank Criteria^(16)^. The severity of the disease was assessed using the HY scale; only patients with a score 0-2 were recruited. Moreover, the duration of the disease and the prescribed treatments were recorded. One eye of each patient was randomly selected to avoid bias due to the interrelation between both eyes of individuals.

All procedures were performed according to the tenets of the Declaration of Helsinki. The Ethics Committee of the Miguel Servet University Hospital, Zaragoza, Spain approved the experimental protocol of this study, and all participants provided written informed consent.

A comprehensive ophthalmological evaluation was performed for each subject, including visual acuity, refraction, intraocular pressure (IOP), anterior chamber exploration, optic disc examination, and Humphrey perimetry (SITA Standard 24.2). Patients with a spherical equivalent >5 diopters or astigmatism >3 diopters were excluded from the analysis. Other exclusion criteria were IOP >20 mmHg, media opacifications (score >0 in the Lens Opacities Classification System III), other ophthalmological pathologies (e.g., glaucoma) or surgeries (except for cataract surgery without incidents), and other systemic pathologies that can affect the thickness of the retina or RNFL (i.e., diabetes, dyslipidemia, uncontrolled arterial hypertension, vasculitis, nephropathy, precedent cardiac disease, and neurological diseases [dementia, Alzheimer’s disease, multiple sclerosis, and peripheral nerve disease caused by a pathology other than PD]). Furthermore, eyes with suspicion of glaucomatous damage were excluded from the study.

The Deep Range Imaging Triton SS-OCT device (Topcon, Tokyo, Japan) was utilized to perform structural measurements of the retina and optic nerve. This device uses a tunable laser providing a 1,050-nm wavelength light, reaching a scanning speed of 100,000 A-scans per second, and yielding 8 and 20 µm axial and transverse resolution in tissues, respectively. In this study, the 3D(H) Macula + 5 LineCross protocol (Wide protocol) was utilized. This protocol examines a large retinal area, providing a fast evaluation of the macular and peripapillary areas. It performs a 12.0 × 9.0-mm three-dimensional scan and a double 9.0-mm radial scan of the macular and peripapillary areas. Additionally, it obtains measurements for the nine macular areas of the Early Treatment Diabetic Retinopathy Study (ETDRS) scan, six macular sectors, and 4-12 sectors of peripapillary thickness of the temporal-superior-nasal-inferior-temporal scan (TSNIT scan).

The ETDRS scan determines the full retinal thickness of the nine macular areas (a central 1-mm circle representing the fovea, as well as an 3-mm inner and a 6-mm outer ring), central and average thickness and macular volume, as well as choroidal thickness (from the Bruch membrane to the choroidal-scleral interface)^(17)^. Scanning of the six macular sectors (i.e., superotemporal, superior, superonasal, inferonasal, inferior, and inferotemporal) provides measurements of the ganglion cell layer (GCL) (GCL+, from the RNFL to the boundaries of the inner nuclear layer; and GCL++, from the inner limiting membrane to the boundaries of the inner nuclear layer) and the choroid.

The TSNIT peripapillary scan provides automatic independent measurements of several retinal layers in the peripapillary area: retinal thickness (from the inner limiting membrane to the boundaries of the retinal pigment epithelium), RNFL (from the internal limiting membrane to the boundaries of the GCL), and GCL+ and GCL++ as described earlier in this article. The TSNIT peripapillary scan provides measurements of four quadrants (i.e., temporal, superior, nasal, and inferior), six sectors (i.e., temporal, superotemporal, superonasal, nasal, inferonasal, and inferotemporal), and 12 clock sectors. Automatic calibration software was used to determine the distance between the delimiting lines in the retina and choroid.

All scans were performed by a single, experienced operator. The measurements were carried out in triplicate for each eye with a time interval of 1 minute between measurements. The Deep Range Imaging Triton SS-OCT device displays a quality scale, indicating the strength of the signal. The quality score ranges from 0 (lowest quality) to 100 (highest quality). In our study, only images with a score >55 were analyzed; those with lower quality were rejected prior to data analysis.

All variables were registered in a database produced using a commercial database application program (Excel, Microsoft Office). The commercial predictive analytics software (SPSS, version 20.0; SPSS, Inc., Chicago, IL, USA) was used to perform statistical analysis. The Kolmogorov-Smirnov test was used to confirm the normality of the sample distribution. A p≤0.05 denoted statistical significance for all calculations.

The COV of a parameter was calculated as the standard deviation divided by the mean value of the parameter and multiplied by 100. The COV shown in tables 1 and 2 is the mean value of the COV - calculated by adding the COV of all patients and dividing the result by the total number of patients (63) - and the standard deviation of the COV, for every sector and layer.

**Table 1:** Coefficients of variation (% ± standard deviation) of macular measurements obtained using swept-source optical coherence tomography

ETDRS	Retina	Choroid
Inner temporal	0.29 ± 0.18	1.82 ± 2.05
Inner superior	0.35 ± 0.30	2.97 ± 3.34
Inner nasal	0.23 ± 0.17	2.22 ± 2.40
Inner inferior	0.27 ± 0.28	2.17 ± 3.11
Outer temporal	0.37 ± 0.35	1.38 ± 1.70
Outer superior	0.51 ± 0.51	2.15 ± 2.34
Outer nasal	0.25 ± 0.20	2.05 ± 2.70
Outer inferior	0.29 ± 0.21	1.60 ± 1.73
Average	0.21 ± 0.17	1.18 ± 1.41
Center	1.38 ± 0.78	4.26 ± 4.28
Total volume	0.21 ± 0.17	1.17 ± 1.40
Inner ring	0.28 ± 0.15	2.30 ± 2.06
Outer ring	0.35 ± 0.23	1.80 ± 1.56
Mean	0.40 ± 0.20	2.09 ± 1.77
Six macular sectors	GCL+	Choroid
Total	0.47 ± 0.59	1.17 ± 1.43
Superotemporal	0.76 ± 0.76	2.01 ± 2.78
Superior	1.15 ± 0.72	2.20 ± 2.08
Superonasal	0.79 ± 0.60	2.71 ± 3.77
Inferonasal	0.88 ± 1.01	1.89 ± 1.57
Inferior	0.83 ± 0.54	1.59 ± 1.47
Inferotemporal	0.97 ± 0.77	1.24 ± 0.96
Mean	0.84 ± 0.49	1.83 ± 1.59

ETDRS= early treatment diabetic retinopathy study; GCL= ganglion cell layer GCL+ includes retinal layers from the retinal nerve fiber layer to the boundaries of the inner nuclear layer.

**Table 2:** Coefficients of variation (% ± standard deviation) of the peripapillary measurements obtained using swept-source optical coherence tomography

	Retina	RNFL	GCL+	GCL+ +	Choroid
Basefile	0.32 ± 0.23	1.85 ± 1.79	2.36 ± 1.73	1.12 ± 1.14	2.72 ± 3.32
Total	0.31 ± 0.22	1.62 ± 1.22	1.40 ± 1.07	0.77 ± 0.67	4.32 ± 5.83
*Four quadrants*					
Temporal	0.82 ± 0.65	2.81 ± 2.95	5.75 ± 5.06	1.91 ± 1.95	2.92 ± 2.57
Superior	0.45 ± 0.21	4.32 ± 4.04	3.77 ± 3.03	2.59 ± 2.94	3.42 ± 4.19
Nasal	0.52 ± 0.46	2.36 ± 1.82	6.77 ± 5.37	1.22 ± 0.74	2.42 ± 3.63
Inferior	0.31 ± 0.22	1.62 ± 1.22	1.40 ± 1.07	0.77 ± 0.67	4.32 ± 5.83
Average	0.52 ± 0.27	2.78 ± 1.93	4.42 ± 2.65	1.62 ± 1.28	3.27 ± 3.20
*Six sectors*					
Temporal	0.75 ± 0.68	2.99 ± 2.31	8.17 ± 4.85	1.73 ± 1.73	3.45 ± 2.69
Superotemporal	1.14 ± 0.87	4.02 ± 3.91	7.19 ± 7.17	2.78 ± 2.60	3.78 ± 3.00
Superonasal	0.40 ± 0.23	3.86 ± 4.00	3.81 ± 2.43	2.25 ± 2.65	3.11 ± 3.75
Nasal	0.88 ± 0.73	3.18 ± 2.16	7.16 ± 5.57	2.00 ± 1.39	3.83 ± 5.24
Inferonasal	0.50 ± 0.58	2.87 ± 2.28	10.28 ± 8.53	1.11 ± 1.04	2.89 ± 3.27
Inferotemporal	0.37 ± 0.27	2.12 ± 1.22	1.90 ± 1.18	0.96 ± 0.68	6.30 ± 1.18
Average	0.67 ± 0.38	3.17 ± 1.89	6.42 ± 3.53	1.81 ± 1.26	3.89 ± 3.47
72 *clock sectors*					
Clock 1	0.64 ± 0.41	8.76 ± 8.89	9.24 ± 7.28	3.15 ± 3.03	5.32 ± 5.47
Clock 2	0.58 ± 0.51	2.86 ± 2.74	6.25 ± 12.08	2.40 ± 3.67	3.50 ± 4.68
Clock 3	0.74 ± 0.60	4.44 ± 4.03	6.79 ± 5.22	3.66 ± 3.19	4.10 ± 5.57
Clock 4	0.78 ± 0.58	3.76 ± 2.73	8.02 ± 4.70	2.16 ± 1.27	3.95 ± 4.94
Clock 5	0.90 ± 0.73	2.61 ± 2.05	9.79 ± 7.30	1.96 ± 1.18	4.08 ± 4.84
Clock 6	0.57 ± 0.59	3.17 ± 2.60	11.01 ± 9.44	1.20 ± 1.22	3.02 ± 3.40
Clock 7	0.45 ± 0.24	2.42 ± 1.81	2.61 ± 2.82	1.31 ± 1.10	2.92 ± 4.08
Clock 8	0.54 ± 0.42	2.92 ± 1.67	2.76 ± 1.63	1.12 ± 0.78	8.36 ± 1.46
Clock 9	0.57 ± 0.37	2.75 ± 2.53	2.22 ± 1.55	1.55 ± 1.30	6.03 ± 6.75
Clock 10	0.67 ± 0.58	3.31 ± 2.44	8.89 ± 5.88	1.75 ± 1.71	3.39 ± 2.47
Clock 11	1.35 ± 1.13	4.53 ± 3.97	7.93 ± 6.54	3.32 ± 2.97	4.39 ± 4.19
Clock 12	0.91 ± 0.66	5.10 ± 5.77	9.97 ± 7.39	2.52 ± 2.59	3.13 ± 2.81
Average	0.73 ± 0.32	3.89 ± 2.12	7.12 ± 3.38	2.17 ± 1.48	4.35 ± 3.25

RNFL= retinal nerve fiber layer; GCL= ganglion cell layer.

GCL+ includes retinal layers from the RNFL to the boundaries of the inner nuclear layer.

GCL++ includes retinal layers from the inner limiting membrane to the boundaries of the inner nuclear layer.

## RESULTS

A total of 63 eyes of 63 PD patients were included in the study. The male/female ratio was 1:1 (i.e., 31 males and 32 females). The mean age was 70.53 years (range: 49-88 years). The mean best corrected visual acuity was 0.75 in the Snellen scale (range: 0.5-1.1). The mean IOP was 14.09 mmHg (range: 11-19 mmHg). The mean duration of PD was 3.10 years (range: 1-9). Last, the mean HY score was 1.21 (range: 0-2).

Tables 3 and 4 display macular and peripapillary thickness of every area obtained with Triton OCT areas.

**Table 3:** Macular measurements (thickness ± standard deviation) obtained using swept-source optical coherence tomography in the different ETDRS areas and six macular sectors

Macular ETDRS (µm)	Retina	Choroid
Center	240.86 ± 31.70	248.41 ± 87.65
Inner temporal	293.82 ± 23.33	246.27 ± 81.22
Inner superior	305.36 ± 20.11	257.72 ± 86.15
Inner nasal	307.01 ± 25.13	232.13 ± 94.03
Inner inferior	304.54 ± 21.19	238.61 ± 92.90
Outer temporal	248.97 ± 16.47	228.90 ± 70.03
Outer superior	262.63 ± 15.99	246.71 ± 80.09
Outer nasal	280.55 ± 16.60	188.59 ± 88.98
Outer inferior	254.19 ± 16.34	222.31 ± 85.84
Average thickness	270.21 ± 16.29	227.32 ± 78.99
Center thickness	194.57 ± 28.42	246.95 ± 87.76
Total volume	7.63 ± 0.46	6.42 ± 2.23
Six macular sectors (µm)	GCL+	Choroid
Total	68.63 ± 7.30	227.48 ± 78.46
Superotemporal	67.79 ± 7.81	240.23 ± 71.55
Superior	67.60 ± 7.37	255.59 ± 81.48
Superonasal	71.00 ± 7.29	211.82 ± 88.00
Inferonasal	69.73 ± 7.59	199.75 ± 92.13
Inferior	65.72 ± 7.62	228.93 ± 86.94
Inferotemporal	69.75 ± 8.94	228.41 ± 76.03

ETDRS= early treatment diabetic retinopathy study; GCL= ganglion cell layer. GCL+ includes retinal layers from the retinal nerve fiber layer to the boundaries of the inner nuclear layer.

**Table 4:** Peripapillary area measurements (thickness ± standard deviation) obtained using swept-source optical coherence tomography in the four quadrants, six sectors, and 12 clock sectors

	Retina	RNFL	GCL+	GCL+ +	Choroid
Total (µm)	282.86 ± 18.31	99.22 ± 12.18	43.03 ± 5.78	142.26 ± 14.98	140.57 ± 61.17
*Four quadrants (µm)*					
Temporal	274.04 ± 17.89	73.42 ±11.66	51.37 ± 8.67	124.80 ± 12.16	148.24 ± 73.05
Superior	299.71 ± 24.75	118.76 ± 20.12	40.98 ± 7.07	159.75 ± 22.51	155.03 ± 64.25
Nasal	256.87 ± 18.75	76.82 ± 15.55	40.24 ± 6.09	117.06 ± 16.69	142.65 ± 53.81
Inferior	300.88 ± 22.26	127.95 ± 19.12	39.52 ± 6.63	167.48 ± 20.04	116.30 ± 63.60
*Six sectors (µm)*					
Temporal	274.04 ± 17.89	73.42 ±11.66	51.37 ± 8.67	124.80 ± 12.16	148.24 ± 73.05
Superotemporal	311.87 ± 27.18	132.24 ± 22.80	39.68 ± 7.80	171.92 ± 24.95	154.12 ± 66.40
Superonasal	291.28 ± 27.92	108.99 ± 24.87	41.94 ± 8.07	150.93 ± 27.05	156.90 ± 64.98
Nasal	260.87 ± 18.69	81.43 ± 16.08	40.20 ± 6.05	121.64 ± 16.90	142.01 ± 54.23
Inferonasal	295.45 ± 25.03	125.34 ± 24.45	37.99 ± 6.42	163.33 ± 23.87	115.28 ± 61.62
Inferotemporal	313.30 ± 26.07	137.45 ± 23.71	41.50 ± 9.79	178.96 ± 25.58	114.66 ± 69.27
72 *clock sectors (µm)*					
Clock 1	285.51 ± 26.02	103.89 ± 25.09	41.86 ±11.61	145.75 ± 26.11	153.79 ± 62.85
Clock 2	270.15 ± 23.77	87.41 ± 24.58	44.04 ± 9.93	131.45 ± 23.09	150.79 ± 58.54
Clock 3	246.20 ± 16.72	66.15 ± 12.55	39.27 ± 6.84	105.43 ± 14.80	141.36 ± 52.22
Clock 4	254.25 ± 20.67	76.89 ±16.16	37.41 ± 7.10	114.30 ± 18.24	135.80 ± 56.38
Clock 5	279.55 ± 21.51	108.10 ± 21.50	38.46 ± 7.43	146.56 ± 20.41	121.96 ± 59.60
Clock 6	312.26 ± 27.02	142.88 ± 26.64	37.42 ± 7.87	180.30 ± 25.90	110.88 ± 64.67
Clock 7	310.77 ± 27.53	132.82 ± 26.52	42.67 ± 10.69	175.50 ± 27.98	116.07 ± 70.35
Clock 8	273.14 ± 19.11	70.52 ± 14.01	53.02 ± 9.93	123.54 ± 14.96	135.87 ± 75.81
Clock 9	266.58 ± 19.52	62.40 ±11.79	53.84 ± 9.84	116.25 ±11.96	151.04 ± 76.74
Clock 10	282.40 ± 20.28	87.32 ± 15.85	47.25 ± 7.60	134.58 ± 16.96	157.74 ± 72.35
Clock 11	311.38 ± 27.78	130.35 ± 24.54	40.94 ± 8.54	171.29 ± 26.33	152.84 ± 66.02
Clock 12	302.29 ± 30.56	122.17 ± 27.13	40.10 ± 6.96	162.27 ± 28.38	158.41 ± 67.56

RNFL= retinal nerve fiber layer; GCL= ganglion cell layer.

GCL+ includes retinal layers from the RNFL to the boundaries of the inner nuclear layer.

GCL++ includes retinal layers from the inner limiting membrane to the boundaries of the inner nuclear layer.

The reproducibility was very high in all layers of the macular area. The mean COV of the retinal and choroidal thickness in the nine ETDRS areas was 0.40 ± 0.20% and 2.09 ± 1.77%, respectively.

**Figure 1:**
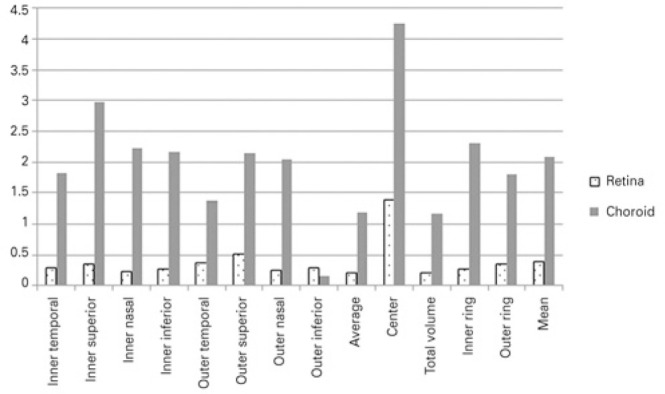
Comparison between the coefficients of variation (%) of retinal and choroidal measurements in the macular area, obtained using swept-source optical coherence tomography

**Figure 2:**
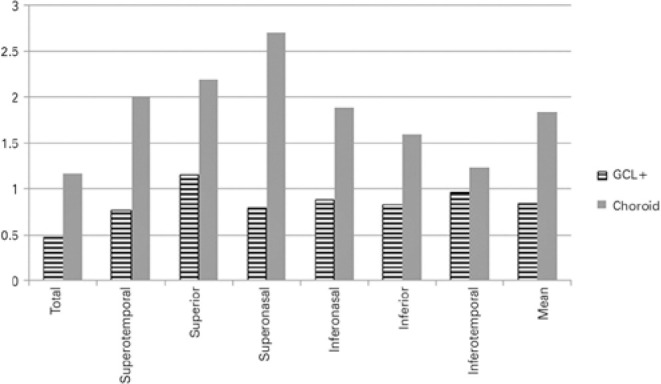
Macular coefficients of variation (%). Data obtained from the scan of the six macular sectors: ganglion cell layer and choroidal.

For retinal thickness, the inner ETDRS ring was more reproducible (COV= 0.28 ± 0.15%) versus the outer ring (COV= 0.35 ± 0.23) (paired-samples t-test, p=0.038). In contrast, for choroidal thickness, the outer ETDRS ring was more reproducible (COV= 1.80 ± 1.56%) versus the inner ring (COV= 2.30 ± 2.06%) (paired-samples t-test, p=0.033). The highest reproducibility of all ETDRS measurements was found in the average retinal thickness and total retinal volume (COV= 0.21 ± 0.17% for both). The lowest reproducibility, though still high, was found in the central choroidal thickness (COV= 4.26 ± 4.28%). Regarding the scan of the six macular sectors, the mean COV of the GCL+ thickness, GCL++ thickness, and choroidal thickness were 0.84 ± 0.49%, 0.57 ± 0.41%, and 1.83 ± 1.59%, respectively. In all three layers, the inferior areas were the most reproducible versus the superior areas which were the least reproducible. The highest reproducibility in the scan of the six macular sectors was found in the total GCL+ thickness (COV= 0.47 ± 0.59%), whereas the lowest reproducibility, though still very high, was found in the choroidal thickness of the superonasal area (COV= 2.71 ± 3.77%) (Table 1, figures 1 and 2).

GCL+= ganglion cell layer.

**Figure 3:**
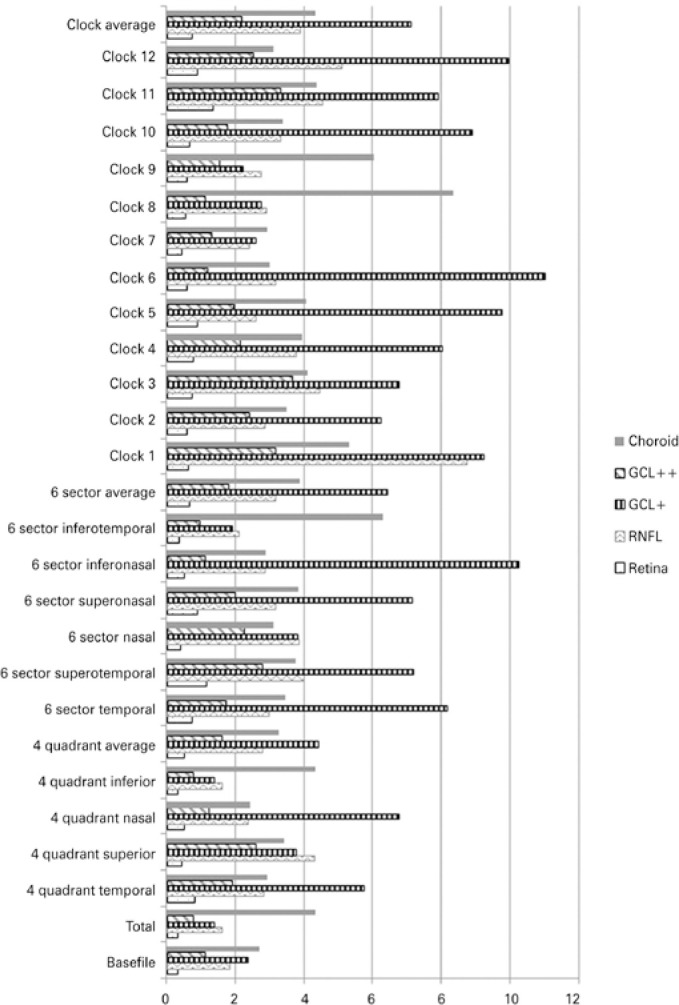
Comparison between the coefficients of variation (%) of the different layers, in the TSNIT scan data (peripapillary area). Data obtained from the four quadrants, six sectors, and 12 clock sectors.

Concerning the peripapillary area, the reproducibility varied depending on the layer and sector. The choroidal thickness showed the highest reproducibility in the nasal quadrant (COV = 2.42 ± 3.63%) and the lowest reproducibility in the eighth clock sector (COV= 8.36 ± 1.46%). The quadrants were more reproducible (COV= 3.27 ± 3.20%) than the six sectors (COV= 3.89 ± 3.47%), while the six sectors were more reproducible than the 12 clock sectors (COV= 4.35 ± 3.25%). The most reproducible area of the GCL+ layer was the inferior quadrant (COV= 1.40 ± 1.07%), whereas the least reproducible area was the sixth clock sector (COV= 11.01 ± 9.44%). The COV of the temporal quadrant, inferotemporal six sectors, and the eighth clock sector were 5.75 ± 5.06%, 1.90 ± 1.18%, and 2.76 ± 1.63%, respectively. The most reproducible area of the GCL++ layer was the inferior quadrant (COV = 0.77 ± 0.67%), whereas the least reproducible area was the eleventh clock sector (COV= 3.32 ± 2.97%). The COV of the temporal quadrant, inferotemporal six sectors, and the eighth clock sector were 1.91 ± 1.95%, 0.96 ± 0.68%, and 1.12 ± 0.78%, respectively. For the full retinal thickness, the most reproducible area was the inferior quadrant (COV= 0.31 ± 0.22%), whereas the least reproducible area was the eleventh clock sector (COV= 1.35 ± 1.13%). The COV of the temporal quadrant, inferotemporal six sectors, and the seventh clock sector were 0.82 ± 0.65%, 0.37 ± 0.27%, and 0.45 ± 0.24%, respectively. The most reproducible area of the RNFL was the inferior quadrant (COV= 1.62 ± 1.22%), whereas the least reproducible area was the first clock sector (COV= 8.76 ± 8.89%). The COV of the temporal quadrant, inferotemporal six sectors, and the seventh clock sector were 2.81 ± 2.95%, 2.12 ± 1.22%, and 2.42 ± 1.81%, respectively. Consistent with the macular scans, the choroidal measurements showed less reproducibility versus retinal measurements in the peripapillary area (Table 2, Figure 3).

RNFL= retinal nerve fiber layer; GCL= ganglion cell layer.

## DISCUSSION

This study assesses the reproducibility of SS-OCT in measuring the thickness of different retinal layers and the choroid in patients with PD. The macular and peripapillary areas were evaluated using the 3D(H) Macula + 5 LineCross protocol, providing information regarding a wide area in a single scan. The most reproducible parameters identified in the present study were the average retinal thickness and total retinal volume in the ETDRS macular scan (COV = 0.21 ± 0.17% for both). Contrary to our expectations, the least reproducible parameter was the sixth clock sector of the GCL+ layer in the TSNIT peripapillary scan (COV= 11.01 ± 9.44%). Of note, previous studies using FD-OCT had shown very high variability in choroidal thickness^(11^,^18)^.

Overall, the reproducibility of SS-OCT was higher in the macular area versus the peripapillary area, in retinal measurements versus choroidal measurements, and in the inferior zones versus the superior zones of the peripapillary area. In the macular area, the choroidal measurements were five-fold more variable versus the retinal measurements (COV 2.09 ± 1.77% vs. COV 0.40 ± 0.20%, respectively). For both retinal and choroidal measurements in the peripapillary area, the reproducibility was positively associated with the size of the field. Thus, the quadrants were more reproducible versus the six sectors, while the six sectors were more reproducible than the 12 clock sectors. For GCL++, total retinal thickness, and RNFL, the inferior and superior zones of the peripapillary area were the most and least reproducible areas, respectively.

For retinal thickness, the inferotemporal zones of the peripapillary region were shown to be the most affected areas in PD. Several studies have demonstrated signi ficant thinning of the RNFL in those areas compared with those observed in healthy controls^(6^-^9)^. A recent study using SS-OCT^(9)^ showed thinning of the inferotemporal sector of the GCL layer in the peripapillary area of PD patients using retinal layer segmentation. This thinning was attributed to the dopaminergic neurodegeneration of retinal ganglion cells^(19)^. In the present study, SS-OCT was shown to be highly reproducible in these sectors (i.e., inferior and temporal).

A number of studies using FD-OCT^(8^,^20)^ have shown thinning of the GCL layer and RNFL in the macular area, and decreased total macular volume in patients with PD. In the present study, SS-OCT was also shown to be highly reproducible in those parameters.

Garcia-Martin tested the reproducibility of two FD-OCT devices for retinal measurements (RNFL) in the peripapillary area of patients with PD^(7)^. In that study, Cirrus OCT yielded a mean COV of 5.38 ± 1.6%, with a lowest COV of 2.10%. The glaucoma protocol of Spectralis OCT yielded a mean COV of 2.35 ± 1.1%, with a lowest COV of 1.03%. Moreover, the Nsite axonal protocol of Spectralis OCT yielded a mean COV of 4.20 ± 2.5%, with a lowest COV of 1.84%. Although these COV indicate a very high reproducibility, they are markedly lower versus the high reproducibility provided by SS-OCT in the present study (COV= 0.21 ± 0.17%).

Currently, information regarding the reproducibility of SS-OCT is limited. Mastropasqua evaluated the reproducibility of measurements of the foveal avascular zone area in 64 eyes of healthy individuals, obtaining COV of 2.44-2.66%^(21)^. Few studies have evaluated the reproducibility of retinal thickness using SS-OCT devices, and none of those reported COV. Our study sheds light on the reproducibility of retinal measurements obtained using SS-OCT in patients with a neurodegenerative disease. FD-OCT is useful for the diagnosis and follow-up of other neurodegenerative diseases (e.g., multiple sclerosis or Alzheimer’s disease), demonstrating thinning of the RNFL, especially in the temporal sector of the peripapillary area^(22^-^24)^. Further studies investigating other neurodegenerative processes are warranted to establish the superiority of SS-OCT over FD-OCT devices in the evaluation of neurological patients.

Vascular parkinsonism has been shown to cause vascular alterations^(25)^. However, in the brains of patients with idiopathic PD, these alterations have not been reported ^(26)^. Recently, Kromer et al. studied the peripapillary retinal vessels using FD-OCT in patients with PD, and observed changes in the morphology of the retinal veins. The investigators suggested that these alterations may be attributed to hypoperfusion, blood speed alterations, or vascular wall modifications^(27)^.

Concerning the reproducibility of choroidal measurements, the currently available studies are based on FD-OCT. To the best of our knowledge, this is the first study investigating the reproducibility of measurements of choroidal thickness using a SS-OCT device. Notably, the results obtained using FD-OCT devices are highly variable. Shao studied the reproducibility of measurements of the subfoveal choroidal thickness through enhanced depth imaging using FD-OCT in 21 individuals without specific pathology. The mean COV obtained in that analysis was 0.85% ± 1.48%^(28)^. However, Karaca et al., evaluated the reproducibility of choroidal measurements in the macular area in 110 healthy individuals using the same approach, obtaining variable COV (24.7635.74%) depending on the examined area^(18)^. In our study, the lowest COV for choroidal thickness was 1.17 ± 1.40%, in the total macular volume. Recently, Satue et al., using SS-OCT, observed choroidal thickening in both the macular and peripapillary areas of patients with PD versus healthy individuals^(9)^. This is a remarkable finding, and inconsistent with previous results obtained using FD-OCT, in which the limits of the choroidal plexus are manually set ^(10)^. This evidence emphasizes the importance of assessing the reproducibility of SS-OCT in healthy individuals and patients with PD.

In conclusion, SS-OCT is highly reproducible for the measurement of retinal and choroidal thickness in both the macular and peripapillary areas. The reproducibility was higher in retinal measurements versus choroidal measurements, in the macular area versus the peripapillary area, in larger sectors of the peripapillary area (i.e., quadrants) versus the smaller sectors (i.e., six sectors and 12 clock sectors), and in the inferior zones versus the superior zones of the peripapillary area. Contrary to our expectations, a sector of the GCL+ layer was the most variable. SS-OCT demonstrated very high reproducibility in the inferior and temporal sectors of the peripapillary area, the most affected zones in neurodegenerative diseases. Further studies evaluating reproducibility in other neurodegenerative diseases are required to confirm the superiority of SS-OCT over FD-OCT. Moreover, additional studies in patients with PD are necessary to corroborate the results of the present investigation.
